# Microarray Embedding/Sectioning for Parallel Analysis of 3D Cell Spheroids

**DOI:** 10.1038/s41598-019-52007-w

**Published:** 2019-11-08

**Authors:** Jonathan Gabriel, David Brennan, Jennifer H. Elisseeff, Vince Beachley

**Affiliations:** 1 Department of Mechanical Engineering, Rowan University, Glassboro, NJ USA; 2 Department of Biomedical Engineering, Rowan University, Glassboro, NJ USA; 3 Translational Tissue Engineering Center, Johns Hopkins School of Medicine, Baltimore, MD USA

**Keywords:** High-throughput screening, High-throughput screening, High-throughput screening, High-throughput screening

## Abstract

Three-dimensional cell spheroid models can be used to predict the effect of drugs and therapeutics and to model tissue development and regeneration. The utility of these models is enhanced by high throughput 3D spheroid culture technologies allowing researchers to efficiently culture numerous spheroids under varied experimental conditions. Detailed analysis of high throughput spheroid culture is much less efficient and generally limited to narrow outputs, such as metabolic viability. We describe a microarray approach that makes traditional histological embedding/sectioning/staining feasible for large 3D cell spheroid sample sets. Detailed methodology to apply this technology is provided. Analysis of the technique validates the potential for efficient histological analysis of up to 96 spheroids in parallel. By integrating high throughput 3D spheroid culture technologies with advanced immunohistochemical techniques, this approach will allow researchers to efficiently probe expression of multiple biomarkers with spatial localization within 3D structures. Quantitative comparison of staining will have improved inter- and intra-experimental reproducibility as multiple samples are collectively processed, stained, and imaged on a single slide.

## Introduction

**Figure 1:**
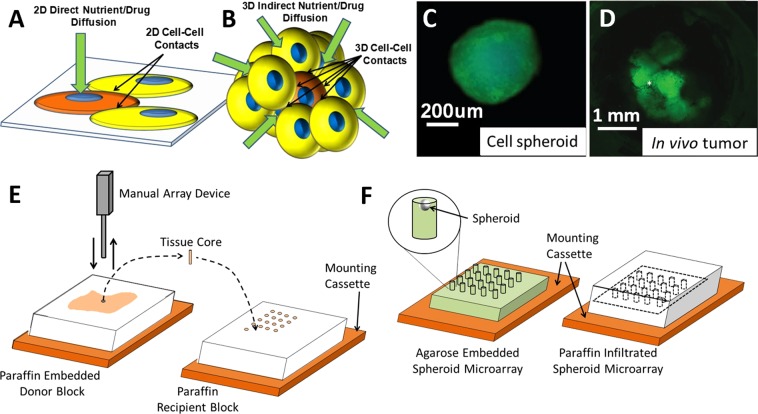
Schematics show (**A**) Perimeter cell-cell contacts and single surface diffusion in 2D. (**B**) Global cell-cell contacts and global diffusion through surrounding cells in 3D. (**C**) The gross appearance of a 3D cell spheroid (live cells stained with calcein-AM) is similar in shape and size to (**D**) structures that make up an *in vivo* breast cancer tumor growing in a mouse^23^. (**E**) Traditional tissue microarray (TMA) uses an array coring device to remove and arrange tissue cores from paraffin embedded tissue samples in a paraffin recipient block. (**F**) The spheroid microarray is arranged in aqueous conditions and stabilized in agarose. The entire microarray and mounting cassette are infiltrated with paraffin to enable sectioning.

Biological systems are extremely complex and are not well represented by conventional two-dimensional cell monolayer culture models. Cells cultured in monolayer adopt a flat/spread morphology with a majority of cellular attachment points to flat substrates on the basal surface of each cell. In this configuration, the diffusion of nutrients and drugs is unrestricted at the apical surface and limited at the basal surface. Cell-cell adherence junctions occur only at the perimeter of morphologically flat cells. These conditions are far from representative of a cell’s normal *in vivo* microenvironment, a three-dimensional arrangement allowing for numerous cell contacts linked by vascular structures and extracellular matrix, promoting the diffusion of oxygen, nutrients, metabolites, hormones, growth factors and cytokines between cells comprising solid tissues, and tumors alike (Fig. 1A,B). Alternatively, cells may be cultured in three-dimensional aggregates using methods such as hanging drop cell spheroid culture to mimic the physiology of *in vivo* systems on a microscale level^1,2^. Cells in three-dimensional configurations adopt the proper shape, experience cell-cell contacts and nutrient diffusion in all directions, and more closely represent the natural microenvironment of tissues and tumors. Nutrient exchange occurs through adjacent cell layers in all planes. Variance in outcomes due to cell culture conditions can be confirmed by measuring drug response characteristics of identical cells cultured in 2D vs. 3D culture systems^3–5^. Thus, 3D cell culture models are important for (1) accurately modeling mechanisms of normal physiological processes, tumor biology and tissue regeneration, and (2) for screening and assessing new treatments^6,7^.

The potential of 3D culture has been recognized by the research community with increased prevalence in journal publications and with the development of many products that facilitate high throughput 3D spheroid culture^8–10^. However, the versatility of these culture systems is limited due to lack of methods of high throughput immunohistochemical biomarker analysis^11^. Histological and immunohistochemical staining identifies and quantifies specific biomarker expression in cellular studies and is easily integrated into arrays of two-dimensional cultures of 96 samples and larger. However, such analysis of 3D cultures requires deep confocal imaging or sectioning and staining of individual samples^12^. Both methods yield valuable biological information, including spatial position within 3D spheroids, but they are inefficient and research costs may be prohibitively high. High throughput 3D *in vitro* models must be compatible with efficient histological and immunohistochemical analysis to realize their potential.

This manuscript describes a straightforward, low-cost microarray technology that improves the efficiency of sectioning/staining/analyzing 3D spheroids. Previously, this method was effectively used to analyze adipose derived stem cell (ASC) response to decellularized tissue particles^13^. The goals of this manuscript are to benchmark performance of the technique, optimize methodologies and to describe the method in careful detail to enable replication and further refinement in other laboratories. Fixed 3D spheroids are arranged in the same plane using a microarray of wells stabilized by an agarose gel matrix for paraffin embedding and sectioning (Fig. 1F). In principle, the methods described share many similarities with tissue microarray (TMA) coring methods currently used to convert tissue biopsy samples into arrays for analysis. TMA coring methods have revolutionized the field of pathology by increasing efficiency and throughput^14^. Traditional microarray procedures use a coring machine to take cylindrical cores as small as 600 μm in diameter, and a few millimeters in length, from paraffin embedded tissue samples. The samples are manually transferred to a paraffin recipient block in a microarray arrangement (Fig. 1E). While this method is not technically impossible for *in vitro* 3D spheroids, their small size makes it extremely difficult to arrange them in the same cutting plane for parallel analysis. Furthermore, traditional TMA requires specialized coring equipment and trained operators. The approaches described within utilize simple equipment allowing for spheroid microarray arrangement under aqueous conditions. Up to 96 spheroids were precisely arranged in a single microarray in the same cutting plane for efficient processing and analysis.

## Methods

### Microarray negative mold fabrication

A negative mold was fabricated to confine spheroids in a microarray pattern. These negative molds can be infiltrated with agarose to make sectionable spheroid microarray paraffin blocks. The negative mold can be made from plastic or polydimethylsiloxane (PDMS). To fabricate the PDMS negative mold, a patterned positive array, which we will call the “pre-mold” was required to produce the final PDMS negative mold. Two approaches for the “pre-mold” were tested: (1) *aluminum pre-mold*: machined from a solid block of aluminum using a computer numerical control machine (CNC) and (2) *agarose pre-mold*: agarose solution was cast in a nylon template. The nylon template was machined using a lathe and drill press.

#### Aluminum pre-mold approach (PDMS)

**Figure 2:**
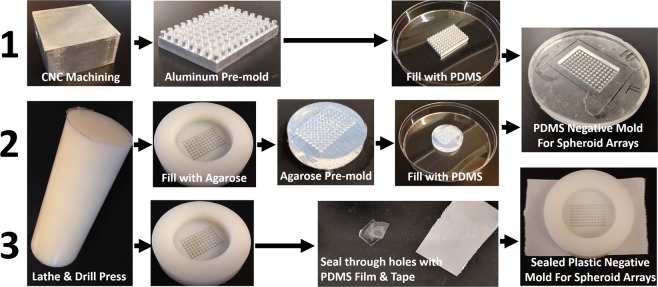
Negative mold fabrication by three alternative methods: (1) Aluminum pre-mold is machined from a solid block of aluminum and immersed in PDMS within a petri dish then removed leaving a PDMS negative mold. (2) Agarose pre-mold is cast in a plastic template, where the template is machined from a rod using a lathe and drill press. The plastic template is filled with agarose solution that is cured to form a hydrogel that is removed and immersed in PDMS within a petri dish. After the PDMS is cured the agarose gel is removed leaving a PDMS negative mold. (3) A plastic mold can be used directly as the negative mold when the through holes are sealed by pressing a PDMS film over the bottom of the mold and securing with tape (Supplemental Video 2).

A positive, aluminum mold was designed in SolidWorks™ (file available, Supplemental Methods S.15) and machined using a 5°, 1/32” tapered end mill (Ford) via a Computer Numeric Control (CNC) process **(**Fig. 2**)**. An end mill with 5° tapers was selected to provide relief during mold release. Posts were made as close as possible within machining limits. For example, final dimensions of a typical aluminum mold were 1.5 mm diameter × 1.5 mm tall posts with a center to center spacing of 2.39 mm between posts in the array. The aluminum part was used along with the Slygard® 184 Silicone Elastomer Kit (base-to-curing agent ratio of 7:1) to create a PDMS negative mold. The silicone mixture was poured over the positive aluminum mold into a 125 mm petri dish and allowed to cure under vacuum at 65 °C for a period of 24 hours to create the final PDMS negative mold. Microarrays generated with this approach were used for analysis presented in this manuscript.

#### Agarose pre-mold approach (PDMS)

**Figure 3:**
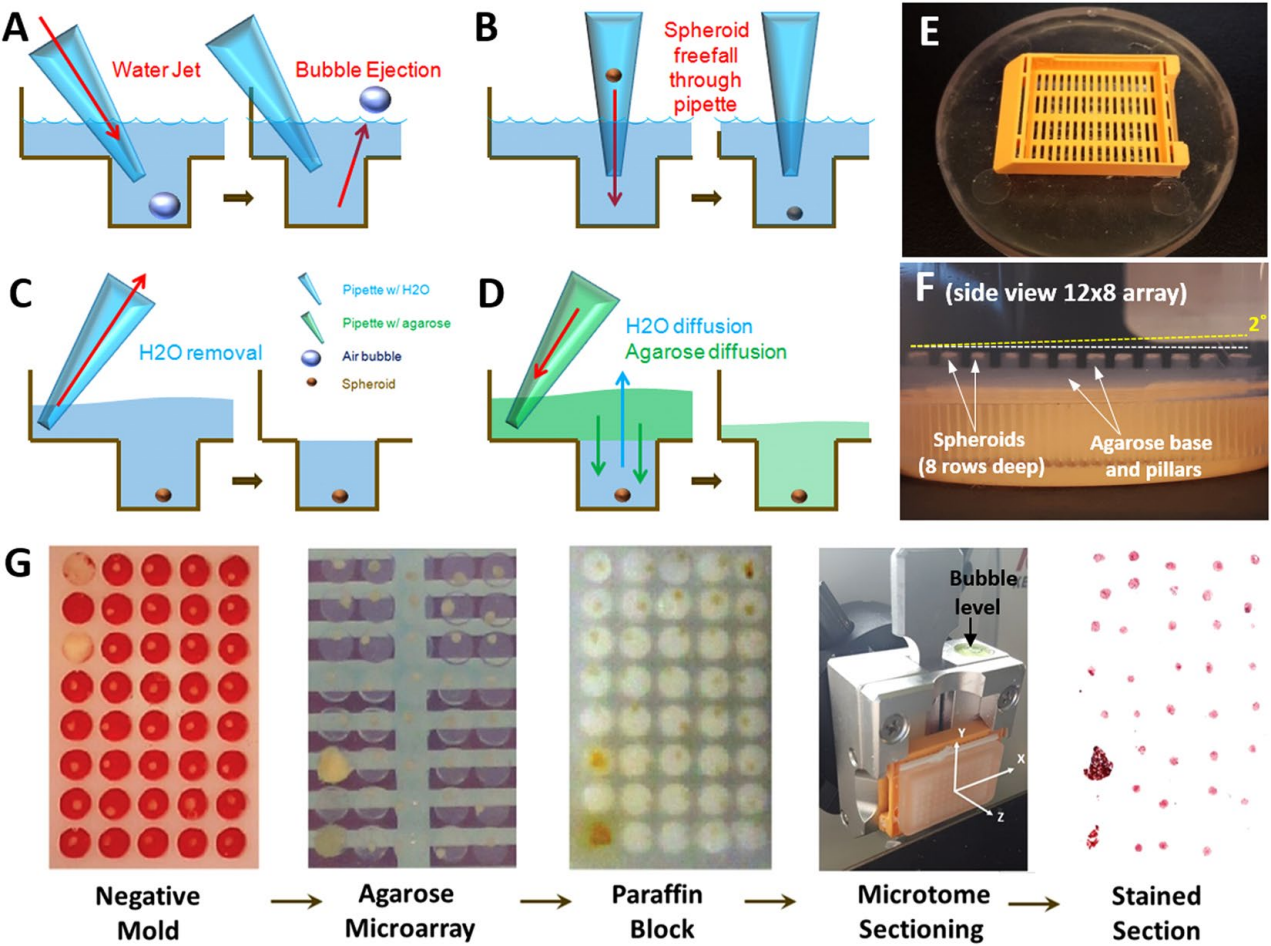
Agarose microarray fabrication (with spheroids). (**A**) Microjetting bubble removal technique. (**B**) Spheroid gravitational transfer technique. (**C**) Water removal. (**D**) Agarose infiltration/mixing. (**E)** Final cassette position on PDMS negative mold. (**F**) 96 spheroids embedded in 12 × 8 agarose microarray attached to sectioning cassette from side view. Spheroids appear to be in the same sectioning plane (white dotted line) and parallel to the cassette base. As a frame of reference, a yellow dotted line shows spheroid offset in the z direction that would occur with a 2° misalignment of the agarose microarray to the cutting plane. (**G**) Images of a single block going through the sequential process starting with spheroid microarray pattered in water (step **B** above) through a stained paraffin section (large tissue fragments are placed in two wells instead of spheroids for easy identification of microarray orientation). Video available in Supplemental Video 1.

The agarose pre-mold approach involves machining a nylon (or other plastic) template that is used to cast a sacrificial agarose pre-mold (Fig. 2). The negative template is machined from plastic rod using a lathe and drill press. For example, a nylon cylindrical rod of diameter 50 mm was cut to a height of ~7 mm, creating a short, wide cylinder (disk). A single hole of 30 mm diameter was lathed to a depth of 5 mm concentrically into the flat edge of the nylon cylinder, thus creating a nylon cup. Through holes with diameters of 1.5 mm were drilled in a microarray pattern into the base of the inner cupped surface using a drill press. The array was created with a spacing of 1.75 mm between the center of each hole in the array; hole depths 2 mm each. This method allows for closer-packing of the holes of the microarray compared to the aluminum pre-mold approach. A thin PDMS film was pressed to the bottom of the template and taped down to water seal the through holes. Then it was filled with 3% wt/v agarose solution to create an agarose pre-mold. The pre-mold is fabricated using the microjetting protocol described in detail in Figure 3 to ensure removal of air bubbles. Care must be taken to ensure that the base of the agarose pre-mold is completely flat and parallel to the face of the microarray pillars. After curing at room temperature, the positive agarose pre-mold was placed in a 125 mm petri dish and covered with PDMS (Slygard, 7:1 base-to-curing agent) and allowed to cure under vacuum at 60 °C for 2 days. The positive agarose pre-mold was broken up and removed from the PDMS mold with small tools and pressured water leaving a PDMS negative mold containing a microarray of wells.

#### Sealed plastic-mold approach

A third approach can be used to make an agarose spheroid microarray using the plastic template as the negative mold (Supplemental Video 2). A plastic mold with through holes, fabricated and sealed as described above, can be directly used as the negative mold according to methods described in Figure 3.

### Spheroid microarray fabrication and processing

The negative microarray mold was filled with deionized water (Fig. 3A,B). Microbubbles were removed using a 1 mL pipette and the following microjetting technique (Fig. 3A). A pipette was depressed before its tip was submerged into the water above the microwells. The push button was then released, and repeatedly depressed and released through small arcs of motion sending microjets of water (~100 µL each) at submerged microbubbles trapped in the array resulting in bubble ejection. Removal of all microbubbles is imperative for successful microarray fabrication. Alternatively, microbubbles may be removed via centrifugation of a water filled mold for 5 minutes at 1500 rpm with a high degree of efficacy.

Cell spheroids (cultured as described in the Supplemental Methods S.1 & S.2) were then placed individually into microwells using a 1 mL pipette and a gravitational transfer technique. First the pipette tip was trimmed at a 45° angle approximately 2–3 mm from its distal end to allow for the passage of ~500 µm spheroids. Spheroids were pipetted up from the 96 well GravityTRAP™ plate and then the pipette tip was submerged above the desired well in the PDMS negative microarray and held stationary in a vertical orientation, without depressing the plunger. This allowed the spheroid to fall out of the modified pipette tip into the appropriate well of the microarray using gravity alone (Fig. 3B). Arrays of 24- (4 × 6) and 96-spheroids (8 × 12) were created using the 96-well PDMS mold. Arrays of 24-spheroids were created using the central wells of the 96-well mold. Spheroid placement was performed using visual inspection and verified using inverted light microscopy (Carl Zeiss Axio Vert.A1).

After the removal of excess water above the top level of the wells (Fig. 3C), verified arrays were infiltrated with agarose (Fig. 3D). UltraPure™ agarose (Invitrogen, 16500500) was added to deionized water at 3% (wt/v) and boiled until homogenous. Agarose concentration was selected based on experiments described in Supplemental Methods S.4. Agarose solution was cooled to 80 °C and used to infiltrate the assembled microarray by pipetting at a rate of approximately 0.1 mL/sec into the corner of the mold **(**Fig. 3D). Agarose was added until a slight meniscus was formed above the height of the open mold. A tissue cassette (Symport) was then mounted as shown in Fig. 3E and additional agarose was added to the top of the cassette so that agarose would surround cassette on the basal and apical surfaces, fixing the cassette to the agarose microarray solidly. The infiltrated mold and cassette were placed in the oven at 65 °C for 5 minutes to reduce the rate of cooling/curing, while promoting mixing of agarose into the bottom of each well. The microarray assembly was then removed and cooled at room temperature for 45 minutes. The completed microarray was carefully removed from the mold by peeling and pulling on the cassette, which was tightly adhered to the agarose microarray (video of preceding procedures available in Supplemental Video 1). The agarose microarray was dehydrated and infiltrated on a mechanical shaker, medium speed, at 37 °C using the following series of 100 mL solution washes (# of washes X duration) in urine specimen containers (Starplex Leakbuster, 120 mL): 50% ethanol (1 × 3 hr.), 70% ethanol (1 × 3 hr.), 85% ethanol (1 × 3 hr.). 95% ethanol (1 × 3 hr.), 100% ethanol (3 × 3 hr. + 1 overnight), HistoClear II (National Diagnostics, 3 × 3 hr.), molten paraffin wax (Fisher Histoplast LP, 5 × 3 hr. at 60 °C). Fluid mixing could be observed at the periphery of the microarray by holding the specimen jar to the light and shaking. Incompletely mixed solutions could be visualized by the refraction of light by fluid boundary layers near micropillars when disturbed. Such solutions were thoroughly shaken by hand and then allowed an additional hour for mixing on the shaker before being advanced to the next wash. A series of images following one spheroid microarray through the embedding & sectioning procedures is shown in Fig. 3G. Analysis of sections taken from this microarray (Fig. 3G) were used to efficiently investigate the effect of decellularized extracellular matrix particles on adipose derived stem cell differentiation in a previous study of 80 spheroid samples^13^.

### Microtome alignment & histological processing

**Figure 4:**
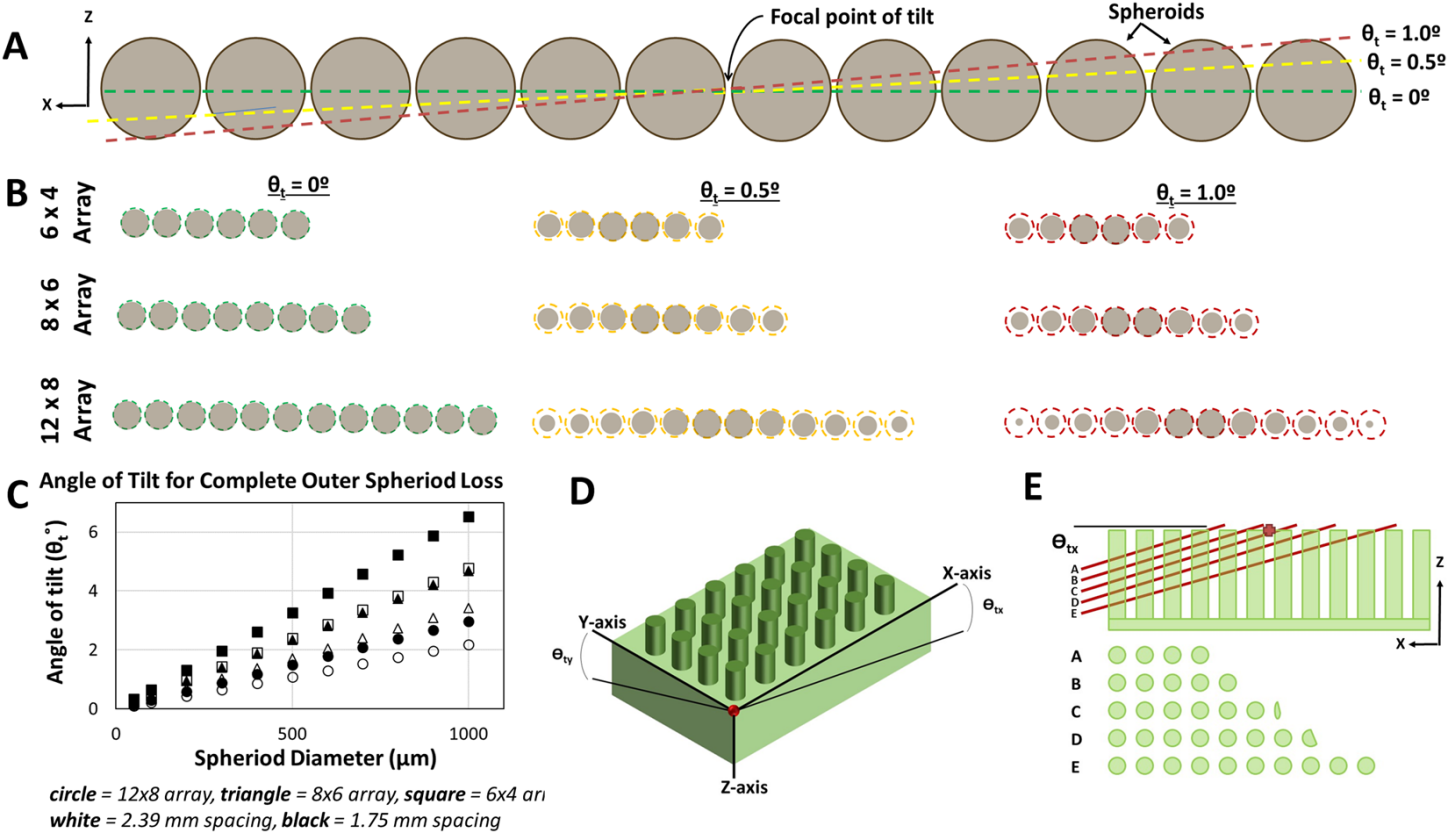
Tilt during sectioning and the relationship of spheroid area on histological sections vs. angle of tilt. (**A**) Schematic model showing the predicted cutting plane through a row of 12 spheroids at three different angles of tilt *ϴ*_t_ = 0, 0.5 & 1°. View is in the xz plane where the y-axis goes through the center of each spheroid in the row. (**B**) The estimated view of the cross-section of the row of spheroids (from **A**) as they would show up on a histological section. The color dotted lines correspond to an “ideal” cross-section through the center of each spheroid. Schematics are shown for 6 × 4, 8 × 6 & 12 × 8 arrays at angles of *ϴ*_t_ = 0, 0.5 & 1°. (**C**) A geometrical calculation predicts the critical angle when the outmost spheroid in an area is completely absent from the centerpoint section. (**D**) The coordinate system and angles of tilt in the xz (*ϴ*_tx_)and yx (*ϴ*_ty_) planes are show against an agarose microarray. (E-top) A schematic of five sequential sections (**A–E**) taken from an agarose microarray paraffin embedded block as viewed from the xz plane. (E-bottom) The corresponding row of pillars appearing on 5 consecutive histological sections used to calculate the experimental tilt for the agarose microarray system.

Paraffin infiltrated agarose spheroid microarrays were mounted in a microtome with three axis of freedom. In order to obtain sections containing the maximum number of spheroids the optimal cutting plane is parallel to the common plane of the spheroid microarray. A procedure described in Supplemental Methods S.5 (Supplemental Video 3) was used to align the cutting plane to the top face of the paraffin block. The angular deviation between the cutting plane and the common plane of the spheroid microarray is referred to as tilt (θ_t_ in Fig. 4A). The critical tilt angle at which all of the spheroids in a microarray could not be obtained on the same slide was estimated using a geometrical calculation described in Supplemental Methods S.6–8. The experimental tilt in practice that was associated with the described procedures was determined by observing the appearance of agarose pillars from different regions of a microarray on consecutive sections (Supplemental Methods S.9). All specimens were taken in 20 µm or 7.5 µm sections, stained using routine H&E with bluing agent (VWR Bluing Agent RTU), and mounted using Fluoro-Gel with TES Buffer (Electron Microscopy Sciences). The effect of processing on spheroid size due to swelling and contraction was quantified as described in Supplemental Methods S.10.

### Quantification of system efficacy

Four independent microarray blocks (n = 4) each containing 24-spheroids were analyzed to determine the quality, quantity and reproducibility of spheroid transfer to histological slides and the effects of processing. The number of spheroids in the array that were successfully transferred to histological sections was quantified using 25% of maximum possible spheroid cross-sectional area as a threshold (Supplemental Methods S.11).

## Results

### Theoretical tilt analysis

Figure 4A shows a visual estimation of where spheroids would be cross-sectioned based on their distance from the center of the array and the angle of tilt. This schematic analysis assumes that all spheroids in the microarray are in the same plane with a spacing of 2.39 mm between each ~500 µm diameter spheroid. The virtual sectioning plane shown in Fig. 4A is from the perspective of the xz plane (defined in Figs 3G and 4D). Three virtual angles of tilt (Θ_*tx*_ = 0, 0.5 & 1°) and three different spheroid microarray sizes (6 × 4, 8 × 6 & 12 × 8) are included in the schematic. The estimated spheroid cross-sections on a resultant histological section are visualized in Fig. 4B with a dotted line representing the spheroid cross-section at an ideal 0° tilt angle. This analysis clearly shows the important inverse relationship between tilt angle and spheroid cross-sectional area on a histological section and the inverse relationship between array size and cross-sectional area of the outermost spheroid in the array. The critical tilt angle where the outermost spheroid is completely missed in a perfect array of 50–1000 µm diameter spheroids is plotted in Fig. 4C (sample calculation shown in Supplemental Methods S.7). Three different size arrays (12 × 8, 8 × 6 & 6 × 4) and two different spacings between spheroids (2.39 mm & 1.75 mm) are plotted. The larger spacing is associated with the aluminum pre-mold method and the smaller spacing is associated with the agarose pre-mold method. The critical angle of tilt varies from 6.51° for 1000 µm spheroids in a compact array to 0.109° for 50 µm spheroids in a wider array. To keep the percentage of outer spheroid area section cross-sectional area to >90% of maximum possible, the critical angle in Fig. 4C for a spheroid of ~25% diameter of the spheroid of interest could be used as a rough estimate. For example, the critical angle for a 125 µm spheroid in a 12 × 8 array with 2.39 mm spacing is 0.272°. A 125 µm spheroid diameter is 25% as large as a 500 µm diameter so one could estimate that the outer spheroid in a 12 × 8 array with 500 um spheroids and 2.39 mm spacing would have >90% of the maximum possible cross-sectional area on a centered histological section for a tilt angle of 0.272° (sample calculation shown in Supplemental Methods S.8).

### Experimental tilt analysis

Figure 4D,E shows how the tilt value is experimentally related to the number of agarose pillars occurring in consecutive sections. In Fig. 4E, A, B, C, D & E represent a single row of agarose pillars showing up on consecutive histological sections. As subsequent sections are taken, the number of agarose pillars on the section changes due to the depth of the cut and the angle of tilt. The number of agarose pillars on consecutive sections were counted to determine experimental tilt associated with the method (Supplemental Methods S.9) Average tilt along the x-axis (θ_tx_) was determined to be 0.115°, correlating to a predicted <2% cross-sectional area lost for the outermost spheroid in a 12 × 8 microarray of 500 µm spheroids. Tilt along the y-axis (θ_ty_) was slightly greater at 0.154° corresponding to a predicted <1% area lost for the outermost spheroid in a 12 × 8 microarray of 500 µm spheroids. These results indicate that spheroid microarray fabrication, mounting and sectioning methods allowed for very accurate alignment of the plane of the agarose microarray to the sectioning plane (xy).

### Processing effects

Spheroids are subject to dehydration and elevated temperatures during processing. Swelling or shrinking of spheroids during processing could affect subsequent sectioning and analysis so these effects were quantified. For a 40 spheroid sample set, the pre-processing maximum spheroid cross-sectional area, measured in PBS after paraformaldehyde fixation, was 1.728 × 10^5^ µm^2^ (σ = 2.270 × 10^4^, n = 40) and the post-processing maximum cross-sectional area, measured on unstained histological sections, was 1.312 × 10^5^ µm^2^ (σ = 7.021 × 10^3^, n = 37). This indicates an average 24.1% reduction in spheroid cross-sectional area and a 13.0% reduction in spheroid diameter associated with dehydration and embedding processing effects. Student’s t-test indicated that this result was statistically significant (p = 1.594 × 10^–9^ < 0.001).

### Efficiency of spheroid microarray histological processing

Spheroid microarray histology will be most powerful when the maximum number of spheroids appear on a single section for parallel analysis and when a maximum number of these ideal sections can be captured for staining for different markers on multiple slides. Thus, two important metrics associated with sectioning were analyzed: (1) total number of histological sections that each individual spheroid appears in within a series of consecutive sections and (2) number of different spheroids appearing on a single section for parallel analysis.

#### Individual spheroid series

**Figure 5:**
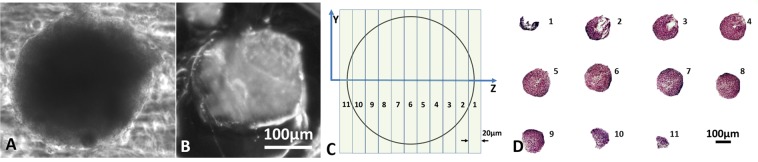
Spheroid (HTB-126) processing effects and sectioning (**A**) Brightfield image of spheroid in PBS after fixation (**B**) Same spheroid after processing and embedding in a microarray in paraffin section. (**C**) Schematic of consecutive sections taken from a single spheroid using the coordinate system in Fig. 3G. (**D**) The cross-section of a single spheroid (stained with H&E) appearing on eleven consecutive 20 µm sections are digitally stitched together in a single image and labeled consecutively.

An image of a spheroid is shown in PBS after paraformaldehyde fixation, but before agarose and paraffin processing and embedding (Fig. 5A ~250 µm diameter**)** and after sectioning, but prior to rehydration and staining (Fig. 5B**)**. The theoretical number of sections that can be taken from this spheroid and the cross-sectional area appearing on each section can be estimated using the spheroid diameter and the section thickness (Fig. 5C**)**. The cross-sections of this single spheroid followed over a series of eleven consecutive sections are shown together in Fig. 5D (selected zoomed in images shown in Supplemental Methods S.12). The thickness of each section was 20 μm. Therefore, eleven consecutive sections represent a total depth of ~220 μm for the series of sections shown in Fig. 5D. This agrees with the size of the spheroid measured after processing (Fig. 5B). Serial sections such as those shown in Fig. 5D are important for analyzing specific regions of a spheroid or to create 3D reconstructions.

#### Microarray sections

**Figure 6:**
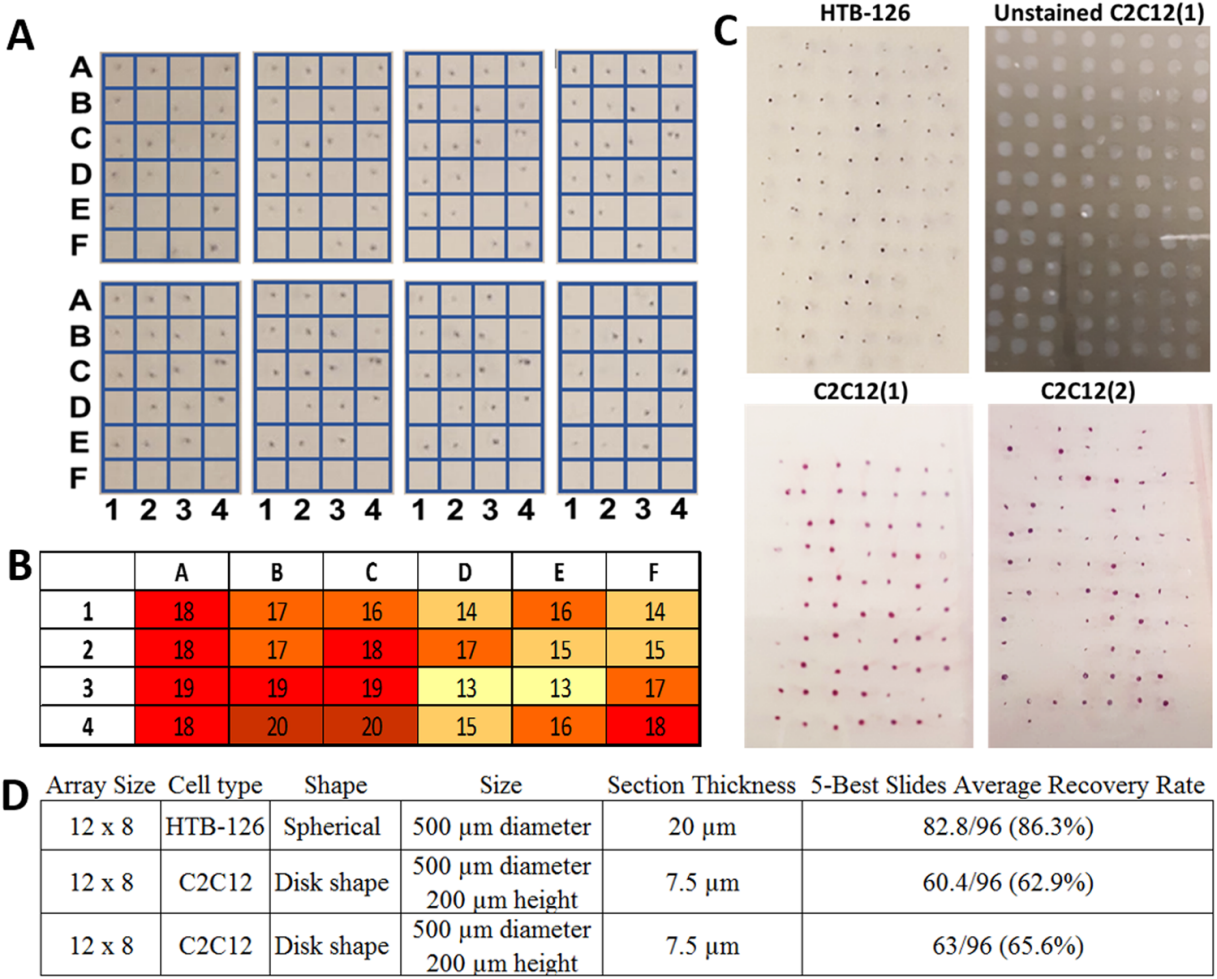
Microarray sections and analysis. (**A**) Eight 20 µm serial sections taken from a 6 × 4 spheroid array (ordered 10, 9, 8, 7, 5, 3, 2, 1; from top left to bottom right; sections 4 & 6 were broken or folded during processing and unusable) from a 24-spheroid microarray. (**B**) Heat map containing number of times a spheroid was recaptured from a respective well in the 5 best sections of four different 24-spheroid microarrays (max possible = 20). (**C**) One section from each of three different 96-spheroid arrays are shown respectively from (top left to bottom right): HTB-126 stained with H&E, C2C12(block 1) unstained section with agarose pillars visible, C2C12(block 1) stained with H&E, C2C12(block 2) stained with H&E. (**D**) The table describes each of three 96 spheroid array (12 × 8) blocks and the subsequent sectioned slides. Recovery rate is the number of spheroids appearing on a single slide.

The ability to capture a certain percentage of embedded spheroids on a single slide using agarose microarray methods was analyzed quantitatively for 6 × 4 spheroid arrays, and qualitatively for a 12 × 8 array. For quantitative analysis four 6 × 4 microarrays containing ~500 µm diameter (HTB-126) spheroids were sectioned and the total number of spheroids and their locations within the array were counted. Figure 6A shows eight 20 µm H&E stained sections taken from a single 6 × 4 array at zero magnification (note, two spheroids were purposely placed in well C4 to serve as a reference point to orient sections). Qualitatively, it may be noted that 22 of 24 spheroids in the array appear on at least four of the sections and that some spheroids are more prominent at the beginning of the series, while others are more prominent at the end. The average percentage of spheroids appearing on each of the five best consecutive sections (selected and counted as described in Supplemental Methods S.11) of each of four blocks (n = 4) was 79.2, 80.8, 86.7 & 90.0%, resulting in an overall average recovery of 20.2/24 + /−1.2 spheroids per section or 84.2% (total of 404 out of 480 possible: 4 blocks * 24 spheroids per block * 5 sections per block = 480). Figure 6B is a heat map that represents spheroid recovery based on initial location in the microarray. The numbers in Fig. 6B represent the total number of spheroids recovered from an individual location in the microarray on the best five sections from each of the four 6 × 4 spheroid blocks analyzed (maximum possible well score = 20, four blocks * five sections per block). The overall number of spheroids captured in each row, column, and individual well of the array was compiled to determine whether spheroid loss was more likely in areas such as the perimeter or corners of the microarray. ANOVA statistical analysis comparing recapture rates between rows, columns, and individual wells did not identify any systematic loss of spheroids associated with different regions or wells. No single row (p = 0.48), column (p = 0.25) or well (p = 0.28) was found to be recaptured at a rate different than any other. As a proof of concept for using this method with much larger arrays, images of sections from 12 × 8 microarrays containing different types of spheroids and the metrics of spheroid recovery were compiled as shown in Fig. 6C,D and described in Supplemental Methods S.13. One array was constructed with breast cancer cell (HTB-126) spheroids with ~500 µm diameter. Two other arrays were constructed with myoblast cell (C2C12) spheroids with a disc shape ~500 µm in diameter and ~200 µm in height. The average number of spheroids appearing (minimum cross-sectional area >25% of center cut) on the 5 best slides for each block ranged from 62.9 to 86.3%.

## Discussion

Three-dimensional spheroid models have been established as a superior biological representation of *in vivo* tumors compared to traditional 2D methods. Thus, tumor spheroid models become a valuable tool for studying cancer biology and screening drugs. These developments have led to increased research efforts as well as the formation of several companies that market lab wares for high throughput 3D culture. However, these systems are generally only assessable to colorimetric outputs for viability/proliferation and there are no available methods for detailed biomarker staining of spheroids in high throughput. This limitation becomes increasingly important as more complex anticancer drugs are developed that target specific pathways (other than cell death) to control cancer progression and metastasis^15–18^. Agarose spheroid microarray approaches make sectioning/staining procedures assessable to 3D tumor models by increasing throughput by ~96 times. Previous studies have shown that agarose embedding does not disrupt biomarker staining^19–22^. This increase in efficiency leads directly to major reductions in time and cost of labor and reagents for studies. Thus, with the help of this tool, researchers can conduct previously unreasonable 3D tumor model studies with more groups, time points, treatment groups, and replicates or extend the technology to personalized medicine approaches. Furthermore, staining comparison between samples within the same microarray slide will be more consistent because all samples are processed, stained, and imaged in parallel. Researchers will also be able to retrieve spatial localization of biomarker distribution to investigate regional effects associated with drug and nutrient diffusion and hypoxic effects.

The benefits of spheroid microarray embedding/sectioning include

- Increased throughput up to 96 spheroids in one block
- More consistent staining comparison between samples on the same slide
- Spatial localization of biomarker distribution

The goal of this manuscript is to disseminate this tool to researchers around the world to encourage them to conduct high throughput experiments with 3D tumor models at an expansive level that is not currently feasible. We expected that the method is quite transferable because several operators with limited histology experience were trained in the technique and generally could perform it with limited repetition. Three methods to manufacture the negative molds are described to adapt to specific laboratory’s resources, approaches and goals. Fabrication of the microarray mold using an aluminum positive pre-mold has advantages including a single step procedure to make molds, and limited variation between PDMS molds; however, this method involved a more expensive/difficult machining process and presents limitations in array hole-to-hole spacing due to required tooling for fabrication of the positive aluminum pre-mold. Fabrication using the plastic negative mold or agarose pre-mold process is more accessible and requires less skill and programming to manufacture, allowing many different geometric designs to be tested more rapidly. This method also allows for closer hole placement as it lacks the need to account for tooling clearance. The agarose pre-mold method comes at the cost of potential variability between PDMS molds, and the additional agarose step may introduce leveling issues as the material readily swells and shrinks depending on environmental conditions. One of the most important aspects of this agarose microarray embedding method when compared to other approaches^19,20^ is the fixation of the array directly to the cassette base in the same plane as the base (Fig. 3E,F, Supplemental Video 1). This allows the array to be easily aligned to the sectioning plane by aligning the mounting head of the microtome to the blade. Another advantage is that the spheroids are embedded at the very top of the agarose pillars. It is very easy to visualize agarose pillars on freshly cut sections (Fig. 6C) and thus easy to determine when the agarose pillars first enter the sectioning plane. By extension, the operator can easily determine when they have first reached the spheroid microarray that is positioned at the top of the agarose pillars. This approach resulted in extraordinary repeatability when considering the small margin of allowable error for microarray-to-cutting plane alignment (Fig. 4).

The results presented demonstrate the utility of the method by demonstrating multiple (5) histological slides containing more than 85% of initially embedded spheroids in a single slide, including a large 96 spheroid array. Other attempts to section a 96 spheroids array contained less embedded spheroids on a single slide (64%), possibly because the height of these spheroids was smaller or because the HTB-126 and C2C12 spheroid array analysis experiments were performed by different researchers at different time periods. Even if multiple sections must be stained and imaged to capture all 96 spheroid that make up an array, there is still a significant time savings compared to single spheroid embedding/sectioning. Further, since so many samples can be processed simultaneously, some complete sample loss can be accounted for by adding additional replicates. However, we do predict that the process can be further optimized to approach 100% spheroid recovery on several consecutive slides. Design considerations for optimization would include pre-mold fabrication, agarose type, concentration & temperature; pillar height, diameter & shape; dehydration & paraffin infiltration processes; sectioning and mounting techniques; and additional methodology considerations (described in detail in Supplemental Methods S.14). Efficiency of the technique could also be greatly improved with a method of automated spheroid placement from culture location directly into appropriate wells of the microarray.

The spheroid microarray technique described facilitates parallel analysis of up to 96 spheroids from a single paraffin block. The technique is simple to implement with tools available to most laboratories. Parallel analysis will make it more feasible for researchers using spheroid models to conduct detailed experiments that require biomarker staining outputs. With access to biomarker staining, researchers will be able perform more comprehensive 3D *in vitro* spheroid studies. These studies will require less cost, time and reagent, making it possible to run experiments with more cellular conditions, treatment types, doses, time points, and replicates. Importantly, immunohistochemical staining can be performed to yield numerous biomarker outputs with spatial localization and double staining capability. In addition, quantitative comparison of staining intensity between samples within a microarray will be more reliable because all samples are processed, stained, and imaged in parallel. These capabilities and reduced costs will help researchers to advance our understanding of tumor biology and facilitate advanced screening of tumor response to new experimental treatments or personalized medicine approaches. Spheroid microarray approach will also be valuable in applications including developmental biology, drug toxicity, tissue engineering and organ on a chip.

## Supplementary information

Supplemental Methods

Supplemental Video 1

Supplemental Video 2

Supplemental Video 3

## Data Availability

All data described in this manuscript and all details of methods described will be made readily available upon requests made to the corresponding author.
