# Supplementary material for: Microarray Embedding/Sectioning for Parallel Analysis of 3D Cell Spheroids
Source: Sci Rep. 2019 Nov 8;9:16287. doi: 10.1038/s41598-019-52007-w (PMC6841729; doi:10.1038/s41598-019-52007-w)
Supplement: Supplementary file 1 — Supplemental Methods [file 41598_2019_52007_MOESM1_ESM.pdf]

Microarray Embedding/Sectioning for Parallel Analysis of 3D Cell Spheroids

Jonathan Gabriel [1], David Brennan [2], Jennifer H. Elisseeff [3], Vince Z. Beachley [2]
[1]Department of Mechanical Engineering, Rowan University, Glassboro, NJ
[2]Department of Biomedical Engineering, Rowan University, Glassboro, NJ
[3]Translational Tissue Engineering Center, Johns Hopkins School of Medicine, Baltimore, MD

Supplemental Methods

S.1 HTB-126 Spheroid Culture

Hs 578T (ATCC® HTB-126™) breast cancer cells were subcultured with the following media: Dulbecco's Modified Eagle's Medium (Gibco) supplemented with 10% fetal bovine serum (Gibco), 1% penicillin-streptomycin (Gibco), and 0.01 mg/mL insulin isolated from bovine pancreas (Sigma-Aldrich). Cells were incubated at 37ºC with 5% CO_2 and grown in T75 adherent bottom culture flasks (Sarstedt) until 90% confluence. Cells were harvested by trypsinization, washed, and resuspended in fresh media supplemented with 0.24% methylcellulose (25 cP, Sigma Aldrich). Cells were suspended in 40 µL droplets, using the 96 well GravityPLUS™ hanging drop system (InSphero). Culture plates were wrapped in parafilm to reduce media evaporation. Additionally, 15 mL deionized water or 7.5 ml deionized water and 7.5 ml of PBS was added to the bottom chamber of the 96 well GravityPLUS™ system along with a humidifying pad provided by Insphero. The cell suspension was allowed a total period of 72 hours for spheroid formation with a single media change after 48 hours using a 12-channel multipipettor (Fisherbrand Elite); 15 µL of media was aspirated and 17 µL of fresh media was added to account for slight evaporation; evaporation created a slight, inevitable increase in hypertonicity. Breast cancer cells aggregated and formed 3D cellular spheroids with predominantly spherical and some elliptical geometries.

The final size of spheroids was dependent on initial seeding concentration. Concentrations of $4.0x10^4$ cells/mL (1600 cells/spheroid) created spheroids <200 µm that formed readily. An intermediate cell concentration of 4000 cells/spheroid formed spheroids with diameters of approximately 350-400 µm. Seeding concentrations in hanging-drops greater than $2.0x10^5$ cells/mL (8000 cells/spheroid) created culture environments that failed to properly aggregate. The addition of low viscosity methylcellulose (25 cP, Sigma M6385) to culture medium appeared to help aggregation of spheroids at appropriate cell concentrations.

S.2 C2C12 Spheroid Culture

C2C12 (ATCC ®CRL-1772™) mouse myoblast cells were subcultured in Dulbecco's Modified Eagle's Medium (Lonza 12-604F) supplemented with 10% fetal bovine serum (Gibco 16140071), and 1% penicillin-streptomycin (Gibco 15140-122). T75 adherent bottom culture flasks (Sarstedt) with cells were incubated at 37˚C and 5% CO_2 until 90% confluence. Cells were harvested with trypsin (Corning 25-053-CL), washed, resuspended in media at a concentration of 1.6 million cells/mL and mixed at a 1:1 volume ratio with media supplemented with 2.4 mg/mL methylcellulose (4000cP Sigma Aldrich, M0512) prepared similar to a previous description[1]. The 96 well GravityPLUS™ hanging drop plate (InSphero), was used to suspend cells in 40 µL droplets. A final concentration of 800,000 cell/mL resulted in spheroids with approximately 32,000 cells and a disc shape with a diameter of ~ 500 um and a height of 200 um after 3 days of culture. Media was changed after every two days by adding 20 uL of media containing 2.4 mg/mL methylcellulose to each well with a 12-channel multipipettor and then removing 20 uL of media. The process was repeated twice for each media change. The supplementation of high viscosity methylcellulose in the hanging droplet medium was a critical promoter of spheroid

aggregation. The bottom chamber of the GravityPLUS™ plate contains a humidifying pad provided by InSphero as well as 7.5 mL of deionized water and 7.5 mL phosphate buffer saline (PBS). Culture plates were wrapped in paraffin during incubation to prevent media evaporation.

S.3 Spheroid Fixation

After the 72 hour time point, spheroids were transferred into GravityTRAP™ plates (InSphero) by stacking the GravityPLUS™ plate over the GravityTRAP™ plate as described in manufacturer instructions. The wells of the receiving GravityTRAP™ plate were prefilled with 70 µL of PBS and a volume of 70 µL of PBS was added to the hanging drops in the GravityPLUS™ plate so that the hanging droplet made contact with the fluid in the well below. The spheroid and some fluid from the droplet transferred into the GravityTRAP™ plate receiving well. PBS was removed from the GravityTRAP™ wells containing the spheroid according to manufacturer instructions and 2% paraformaldehyde (PFA) (Electron Microscopy Sciences) was added and allowed to incubate for 30 min - 2 hours. After PFA incubation the spheroids were washed within the GravityTRAP™ plate wells and stored in PBS until they were transferred to a microarray for further fabrication.

S.4 Agarose Embedding Optimization

Agarose solution was then added to the mold and was cured as described in Methods. It was observed that high agarose concentration could result in brittle gels prone to pillar fracture or had missing pillars due to incomplete diffusion of highly viscous agarose solutions into the bottom of wells. Low agarose concentration could result in gel geometries that did not match the dimensions of the negative mold. Concentrations of 0.5%, 1%, 2%, 3%, and 4% agarose (w/v) were tested; 3% agarose (w/v) solution was determined to best ensure the complete gelation of pillars for

microarray formation and limit the probability of pillar fracture during pullout. Agarose microarrays fabricated from 2.5-3% solutions added at 80-90°C were easily removed from the mold and contained the spheroids embedded in robust pillars. Spheroid location in the agarose cylinders was tangential to the top surface in height (z-plane) and at a random location in the radial/angular plane (xy plane). **Figure 3F** shows a microarray of 96 spheroids in the same plane during processing.

S.5 Microtome Alignment & Histological Processing

The angle of the sectioning plane relative to the plane of the spheroid microarray was analyzed and will be referred to as "tilt." The microtome (KEDEE KD-2258) used in these experiments has a head that is adjustable around the x-, y-, and z-axes, and translates forward along the z-axis as each section is taken (***Figure 3G***). The desired orientation of the face of the microarray block is parallel to the xy plane. The bubble level attached to the cassette receiver was used to align the cassette about the x- and z-axes (***Figure 3G***); however, alignment of tilt about the y-axis required visual alignment using an iterative process. A blank cassette was placed into the cassette receiver and the microtome blade was installed. The receiver was then adjusted to an initial setting expected to align in the xy-plane. The receiver was translated forward towards the fixed blade until contact between the blade and blank cassette was made; the blade position was verified against the blank cassette at various values along the z-axis, precisely at the intersection between the blade, and each of the two vertical edges of the orange cassette. This relationship was examined at cassette levels y = 0 (corresponding to the level of the blade; middle), y = 0.5 cm (top), and y = -0.5 cm (bottom), to assure alignment was consistent across the entire xy plane of the cassette. Alignment was considered acceptable when both vertical edges of the blank cassette

met the statically locked blade at the same position during forward (z) translation in all three locations along the y-axis (top, middle, bottom of the cassette). Visual verification of contact was limited in exactness to approximately 50 µm across the width of the cassette. After successful alignment of the blank cassette, the receiver was translated away from the blade, and the cassette containing the microarray was mounted. For the sake of the study, it was assumed that all cassettes were manufactured identically. Alternatively, the blade angle was changed to zero degrees and two blank cassettes were loaded in the microtome with the positioning of the cassette mount free to rotate. The blade assembly was moved forward to press flush with the face of the cassettes. The cassette orientation locks were tightened while the cassette was flush with the blade assembly. Then the cassette position was moved back and the blade angle was changed to 5˚ **(**see **Supplemental Video 3**)

The paraffin sections were place in an oven at 48˚C for an hour to increase adherence to microscope slides (Tru Scientific – TruBond 380). Slides were deparaffinized, hydrated and stained with Hematoxylin and Eosin (H&E) as follows:

	Solution	# of washes X duration
1	Histoclear II	n x 5 minutes (until complete removal of paraffin by visual inspection)
2	100% ethanol	2 x 5 minutes
3	95% ethanol	2 x 5 minutes
4	DI H_20	2 x 5 minutes
5	Hematoxylin (VWR 95057-858)	1 x 20 seconds
6	DI H_20	1 x 4 minutes
7	Bluing agent (VWR 95057-852)	1 x 2 minutes
8	DI H_20	2 x 1 minute
9	95% ethanol	1 x 30 seconds
10	Eosin	1 x 20 seconds
11	100% ethanol	1 x 1 minute
12	DI H_20	1 x minute
13	Cover slip with FluoroGel	indefinite

S.6 Theoretical Tilt Analysis

Optimally, the plane containing the spheroid array in the paraffin block will be exactly aligned to the cutting plane of the microtome (xy). Minimizing tilt (the angle between these two planes; θ_t, in **Figure 4**) is critical to obtaining single histological sections with many spheroids, each of adequate area for analysis. A schematic model (**Figure 4A-B**) of x-axis tilt angle versus spheroid area (with perfect alignment in the z and y-axis) shows how, on a centered histological section, the area ratio of spheroid slices in the outer portions of the array decreases farther from the center point. In order to quantify this relationship for arrays of particular geometries, the critical angle of tilt (for example $\theta_t = 1^\circ$ in **Figure 4A**) that would result in the complete absence of the outermost spheroid in the section containing the center point of the array was estimated using geometrical calculations. For these calculations, the focal point of tilt was the centerpoint of the array and the section thickness was assumed to be zero (**Figure 4A**). At the critical angle of tilt, a single section containing some part of each spheroid in the array is not possible. This critical angle is dependent on the size of the spheroids, the number of spheroids in the array and the spacing between each spheroid. A sample calculation for the critical angle of tilt and the % area in outer spheroid cross-section are shown below.

S.7 Supplemental Calculation 1: Estimating Critical Angle of Tilt to Lose Outermost Spheroid (**Figure 4C**)

Sample calculation is for a 12x8 array in the direction of 12 spheroids in the array. A calculation for a 500 µm spheroid array is used. It is assumed that the focal point of tilt angle is at the center line of the array and directly in between the 6th and 7th spheroid in the array (**Figure 4A**). It is assumed that the spheroid array and the section line is straight and the section has zero thickness.

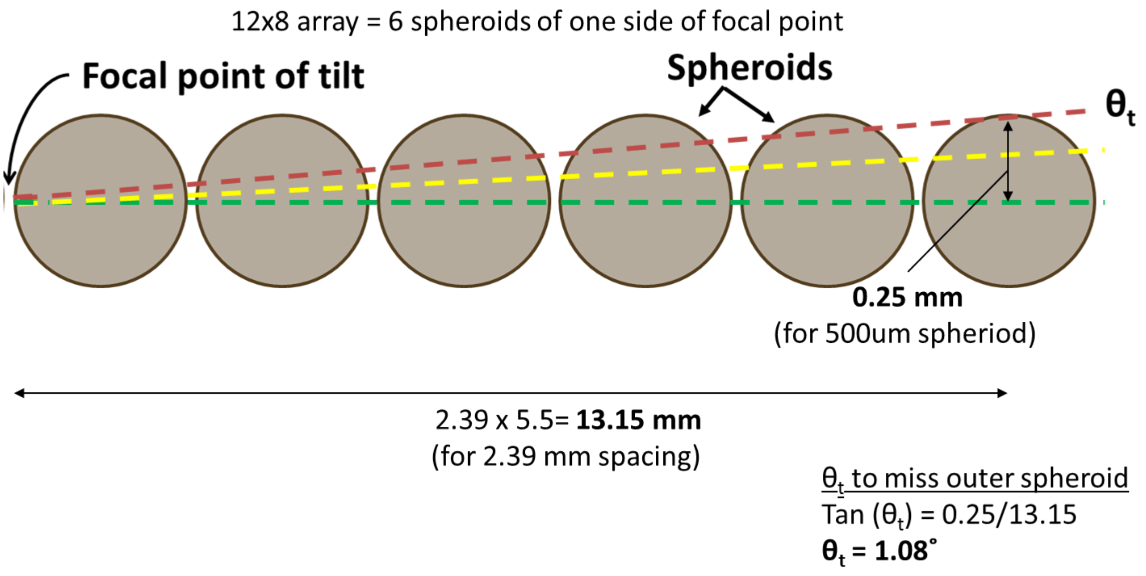

S.8 Supplemental Calculation 2: Estimating Area of Outer Spheroid Transferred to a Section Based on Angle of Tilt

Sample calculation is for the conditions and assumptions listed top left of the figure. First the vertical distance from center of the circle to the cutting plane is estimated. Then the length of the horizontal cross-section through that point is estimated. The radius of that distance is used to provide a rough estimate of the area transferred to a histological slide. Center focal point and array geometry is the same as in **Figure 4A.** Area is presented as a percentage of the area of a horizontal cross-section passing through the center of the circle.

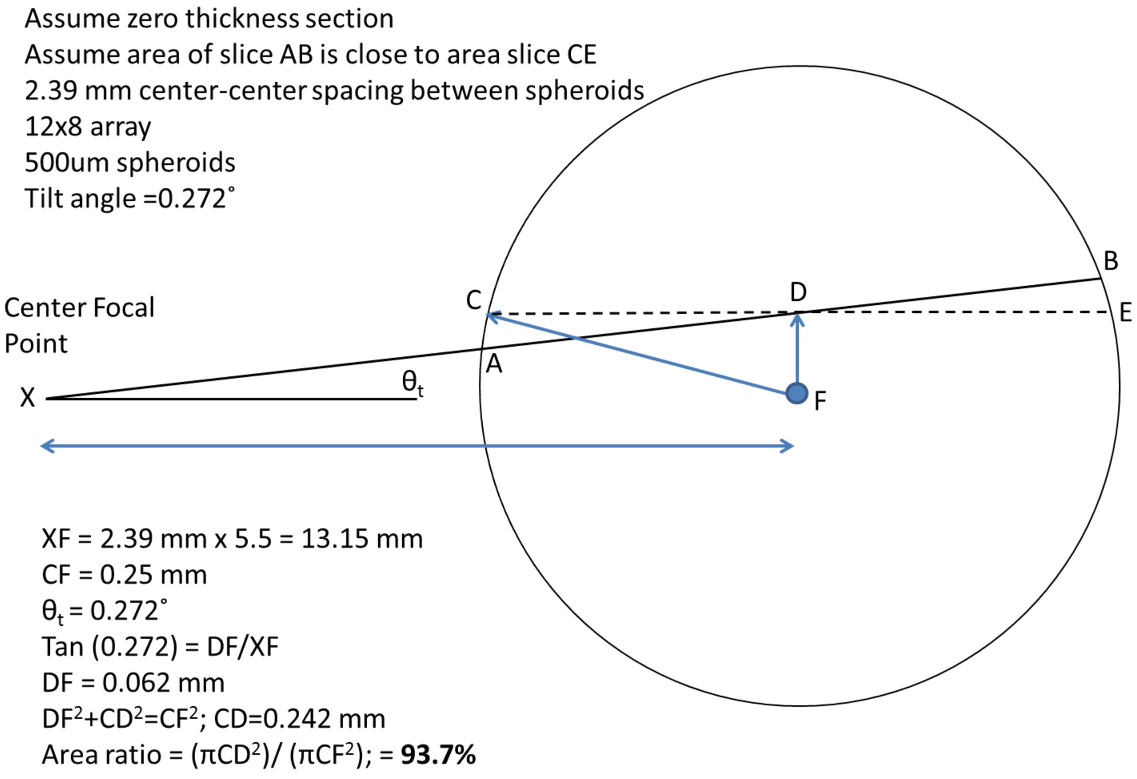

S.9 Experimental Tilt Analysis

To determine the angles of tilt that are associated with the spheroid microarray method in practice, an experimental analysis was conducted using a blank agarose microarray embedded in a paraffin block (**Figure 4D-E**). The plane of a perfect theoretical spheroid array embedded in the agarose microarray was assumed to be parallel to the plane of the tips of the micropillars of the array, offset along the negative z-axis by a value equal to the average spheroid radius, so that the plane passes through the center of each spheroid. Four agarose microarrays blocks (n=4) were sectioned, at a thickness of 20 µm, with 2.39 mm pillar to pillar spacing. Agarose pillars could be observed on the histological slides. Observing and trending blank agarose pillar appearance on serial microarray sections allowed for experimental calculation of the tilt value. The relationship of pillar pattern to tilt angle is depicted by **Figure 4E**, which shows a schematic pattern of how new micropillars of a single row appear in progressive sections in the presence of tilt. Assuming all pillars are in the same plane and of the same length, the tilt can be calculated based on the geometrical arrangement of the pillar microarray on consecutive sections, and the thickness of sections. Independent calculations were made to determine the tilt along the x-axis (θ_{tx}) and the y-axis (θ_{ty}) to measure error associated with aligned the microarray to the cutting plane about different axis.

During sectioning, it was observed that pillars did not always show up uniformly. Often, one side of the block would show micropillar wells before another side. Within a few sections, all pillars would usually become present. Assuming all pillars remained flat through processing, the tilt could be calculated based on vertical displacement (quantified by number of sections) and horizontal displacement (number of new pillars present).

Table S.1 shows the experimentally determined tilt values for 4 different 6x4 arrays. Not all blocks showed such an obvious pattern, and others contained pillars which clearly did not stay flat through processing. Incomplete dehydration of the agarose microarray during initial ethanol washing steps often leads to swelling and the formation of a concave meniscus along the tips of the micropillars.

Table S.1. Experimentally determined tilt.

**Block**	**X-tilt**	**Y-tilt**
B17	0.112º	0.315º
A1	0.116	0.0749
B5	0.0499	0.103
B7	0.180	0.124
**Mean**	**0.115º**	**0.154º**
**Std. Dev.**	**0.0530º**	**0.109º**

Average X-tilt (the long length of the block as described in the tilt section) was 0.115º for a 24-spheroid block, a very low finding. The Y-tilt was slightly greater at 0.154º. The reason for increased tilt angle is the alignment mechanism. The methodology of alignment correlating to X-tilt error involved placing a blank cassette in the microtome and comparing its orientation visually against the blade across its whole length. This method allowed for very accurate alignment. There was no way to replicate this alignment strategy for Y-tilt. Y-tilt was done

strictly using the bubble level attached to the microtome and visual inspection. This explains the larger average error and standard deviation. Overall, tilt values were very low and could not alone account for reductions in the percentage of spheroids recovered.

S.10 Post-Processing Effects on Spheroid Size (Swelling & Contraction)

After fixation in paraformaldehyde, but before agarose infiltration, spheroids were digitally imaged and measured under bright field microscopy while immersed in PBS (**Figure 5A**). Minimum and maximum spheroid diameters were used to estimate the "pre-processing maximum spheroid cross-sectional area" assuming elliptical geometries. This area was deemed the maximum possible cross-sectional area attainable by any single section from the individual spheroid, and theoretically could only be obtained should a section be taken precisely through the center of the spheroid (***Figure 4A***, $\Theta=0°$). After histological processing and prior to H&E staining (**Figure 5B**), minimum and maximum diameters were re-measured for each of ten randomly selected spheroids within a 6x4 array for all sections taken to calculate the cross-sectional area. The largest single value from all sections was called the "post-processing maximum spheroid cross-sectional area" and was compared with the "pre-processing maximum spheroid cross-sectional area" using a t-test. The null hypothesis was that processing had no effect on the mean cross-sectional areas.

S.11 Determining Spheroid Number Per Histological Section

Next, the number of spheroids successfully transferred to slides (termed "recovery") per section was quantified to determine the utility of the process. Four independent spheroid microarray blocks each containing a 6x4 array of ~ 500 μm diameter spheroids were analyzed. Spheroid

recovery was quantified based on a 1/0 score for each spheroid in each well of each section taken from each microarray (n=20). Only spheroids with post-processing cross-sectional areas above the threshold of 25% of the pre-processing maximum spheroid cross-sectional area were scored as a 1, and considered successfully recovered. The procedure for determining recovery (1/0) was the following. First, fixed spheroids in PBS were imaged in wells and measured before processing. Each spheroid was approximated as an ellipse with measurements taken for both the long and short axis of the spheroid. These dimensions were used to determine the "pre-processing average maximum area". The same measurements were used to calculate area from images of stained sections. This meant that spheroid recovery could be unsuccessful due to; (1) loss during sectioning/slide mounting, (2) a spheroid not residing in the same plane (z-coordinate) as the rest of the agarose microarray, or (3) tilt difference between the sectioning plane and the spheroid microarray plane. The total number of successfully recovered spheroids was recorded for each section, and individual well. Each section was scored to pick which sections to use when reporting the percentage of spheroids recovered per block. The score of each section was the number of 10 randomly selected premeasured spheroids (for example, wells: A1, B3, C2, C4, D1, D2, E3, E4, F1, F3) with at least 25% of pre-processing maximum cross-sectional area (as described above) divided by 10. The five consecutive highest scoring sections from each 24-spheroid microarray (n=4) were analyzed, representing a total cutting depth of 100 µm (5 x 20 µm sections) through each spheroid microarray block. ANOVA was performed comparing rows, columns, and individual wells of the microarray to determine whether recovery was dependent on specific regions of the array. The null hypothesis stated recovery rates were the same among spheroid placement in the microarray when comparing a single row from one microarray versus all rows in

all microarrays (n=16), single columns versus all columns (n=24), and individual wells versus all wells (n=120).

S.12 Zoomed in spheroid images from ***Figure 5D***

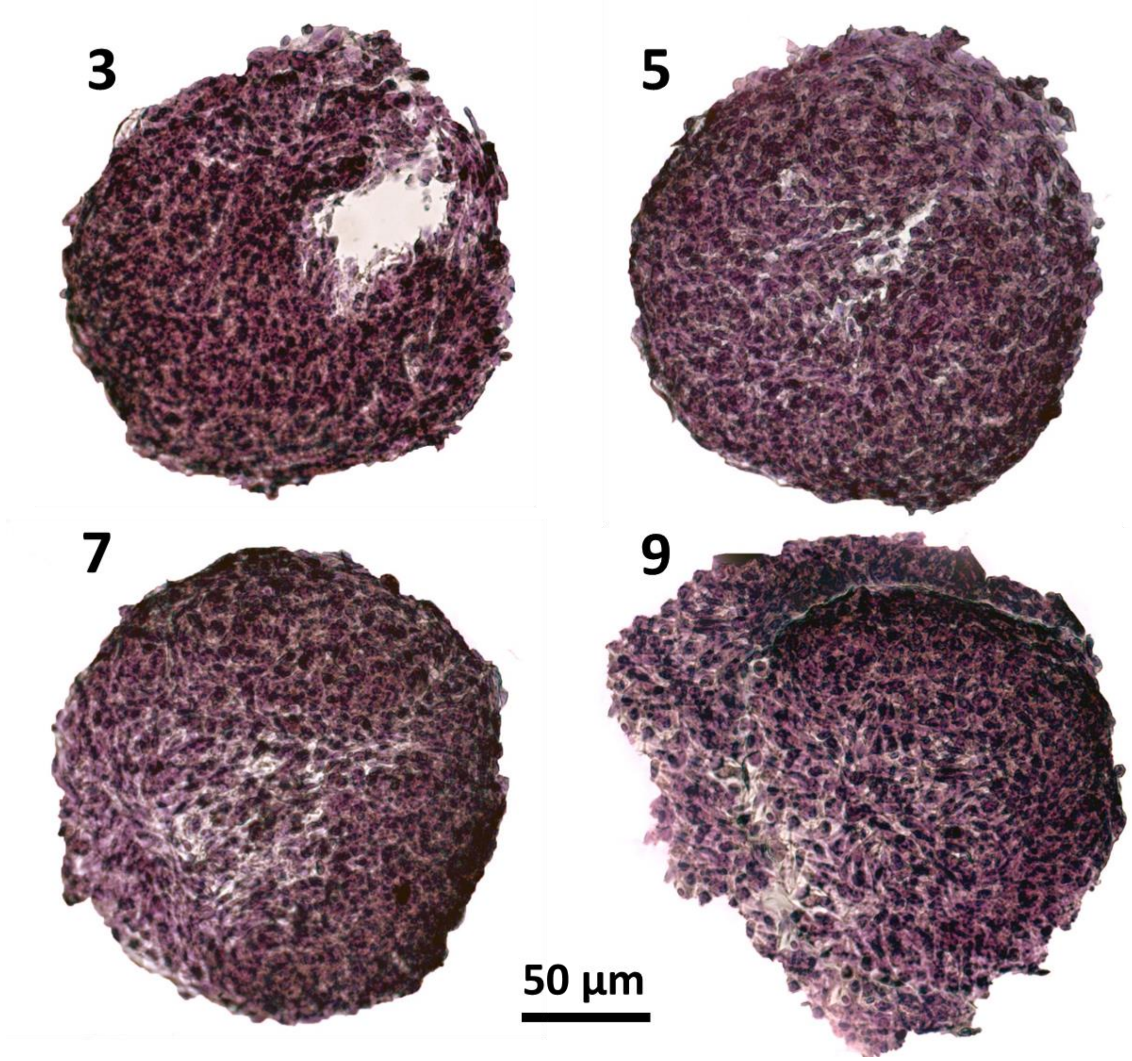

S.13 12 x 8 Microarrays

HTB-126 spheroids were spherical in shape and ~ 500 µm in diameter. C2C12 cells formed disc shaped structures that were ~ 500 µm in diameter and ~ 200 µm in height. Some of the C2C12 spheroids appeared to be positioned with their longest dimension in the xy cutting plane, while

others appeared to be positioned with their longest dimension orthogonal to the xy cutting plane. HTB-126 spheroids were cut in 20 µm sections because this size produced the best histological sections (lack of compression, rolling & tearing) for the operator at the time of sectioning. Every section was taken. A total of 14 sections were analyzed within a 280 µm cutting depth with 13/14 slides containing 48 or more spheroids (50%) and 7/14 slides containing 72 or more spheroids (75%). C2C12 spheroids were cut in 7.5 µm sections for the same reason, approximately one of every 5 sections was taken and stained as possible. A total of 10 sections were analyzed within an ~260 µm cutting depth from each of two blocks with 4/10 & 5/10 containing 58 or more spheroids (60%) and 9/10 & 10/10 containing 38 or more spheroids (40%).

S.14 Areas of focus for optimization

- Completely flat mold – Perhaps the most important feature of this approach is the fixation of the agarose microarray to the mounting cassette in the same plane as the base of the cassette. This allows low angles of tilt and recapture of large numbers of spheroids on a single slide, repeatedly. However, if the dimensions of the pre-molds and molds are not flat then tilt errors will occur. We would recommend verifying the consistent height of the molds, pre-molds and agarose array at various stages of fabrication and processing. This can be performed by taking measurements of the height of the mold or array (cassette base to end of pillar) with a caliper (or ImageJ) at different regions (for example all four corners). This may be especially important for the agarose pre-mold approach since there is an additional step where error could occur.
- Hole size and spacing – Hole size and spacing will determine how easily the array is filled and how many spheroids can be packed into a small area. Hole size will also effect the

tendency of spheroids to be jarred loose from holes when adding agarose. Hole size and depth will affect agarose pull out (limit possibility of pillar fracture) and may affect how well water and agarose mix when the agarose is added to the assembled microarray.

- Agarose & protocol – All data presented here used UltraPure™ agarose (Invitrogen, 16500500). However, we have explored other types of agarose and some may be optimal for specific experiments. Agarose concentration is important for mixing with water in pillars, encapsulation of the spheroids, possible pillar fracture and dehydration. A specific type and concentration of agarose may be ideal for different protocols. The temperature of the agarose when it is added is also critical. Higher temperatures promote complete mixing with water in the pillars (**Figure 3D**), but very high temperature could damage biological samples. In our experiments we looked at several methods of preheating the PDMS mold (briefly or water can evaporate out of the pillars) and keeping the PDMS mold warm after the liquid agarose is added. The latter was generally accomplished by keeping the PDMS mold on a hot plate or moving it to an oven for incubation.
- Dehydration & embedding protocol – Proper dehydration is critical to the success of this method. If dehydration is too fast then the agarose microarray will swell/contract and alter the in-plane geometry that is critical to success. Sufficient time and agitation and visual inspection or dimensional measurement between steps are recommended to determine if swelling/contraction is occurring. Also, the size of the agarose array must be considered. Larger arrays are more prone to contraction/swelling due to larger penetration distances required for reagents. In our experiments, the final overnight 100% ethanol wash was performed with a freshly opened bottle. On one of our samples reported in this manuscript

(**Figure 6C**) there are internal pillars that appear lower the perimeter pillars. This is likely due to some contraction in the center of the microarray.

- Microtome alignment – Microtome alignment is a key component for achieving the greatest number of spheroids per section. We have presented two methods of aligning the microtome to the cutting plane, but this may be dependent on the model of microtome used. A fixed mount (non-adjustable) may be ideal for this approach.
- Plastic negative mold approach (**Figure 2(#3) & Supplemental Video 2**) – This approach may be the easiest route for many labs to use this technique. The plastic negative mold is straightforward to fabricate and spacing between holes can be minimized. Some considerations related to this approach include: (1) *Minimizing burs or machining imperfections*. (2) *Producing a good seal at the bottom of the mold*. We found that a freshly made PDMS film was ideal for sealing. The type of plastic and surface roughness both effect how effective the seal is as well as the surface of the PDMS film. We also found that a high-quality spheroid microarray can be produced even with some slow/limited leaking occurring. (3) *Depositing agarose across the tissue cassette grid*. The agarose level must extend above the tissue cassette grid to firmly attach the agarose microarray to the tissue cassette. If the microarray cavity extends outside of the edge of the tissue cassette or if the outside edge of the mold does not extend beyond the edges of the tissue cassette then agarose can leak out and it may be challenging to fill with agarose to the required height. These problems can be overcome by carefully adding agarose to the center of the tissue cassette or modifying the geometry of the mold.
- Spheroid Transfer – In our experiments were found that spheroid transfer could be more efficient if a small amount of liquid was taken up (as low at 5 µL) so that the spheroid fell

to the tip of the pipette quickly. Flicking the tip of the pipette was an effective method of releasing spheroid that were stuck to the inner surface of the pipette tip so that they would fall to the tip of the pipette with gravity.

S.15 SOLIDWORKS drawing for aluminum pre-mold

Available at:

https://drive.google.com/drive/folders/1iN_N0iRJYlX1WLc5ARyTYb85Q2Z0i98t?usp=sharing

References

1 Naber, H. P., Wiercinska, E., ten Dijke, P. & van Laar, T. J. J. Spheroid assay to measure TGF-β-induced invasion. e3337 (2011).
